# Routine Pulmonary Function Test Can Estimate the Extent of Tuberculous Destroyed Lung

**DOI:** 10.1100/2012/835031

**Published:** 2012-05-01

**Authors:** Eun Joo Lee, Sang Yeub Lee, Kwang Ho In, Se Hwa Yoo, Eun Jeong Choi, Yu Whan Oh, Sanghoon Park

**Affiliations:** ^1^Division of Respiratory and Critical Care Medicine, Department of Internal Medicine, Korea University Anam Hospital, Korea University College of Medicine, Seoul 136-705, Republic of Korea; ^2^Department of Radiology, Korea University Anam Hospital, Korea University College of Medicine, Seoul 136-705, Republic of Korea; ^3^Department of Internal Medicine, Hanil General Hospital, KEPCO Medical Foundation, Seoul 132-703, Republic of Korea

## Abstract

Tuberculous destroyed lung (TDL) is diagnosed by a clear past history of tuberculosis with findings of parenchymal destruction verified by chest X-ray. Despite the resultant deterioration of lung function and quality of lives seen in TDL patients, the exact mechanism or characteristics of pulmonary function worsening have not been clearly studied. We investigated the feature of respiratory impairment of TDL patients, and studied whether extent of destroyed lung measured with chest CT has any correlation with routine lung function. To evaluate the degree of destruction, the Goddard classification scoring system was modified into a novel scoring system (destroyed lung score, (DLS)) with a score from 0 to 4. Twenty-five subjects were enrolled. TDL predominantly manifested as an obstructive pattern (64%, 16/25). Median value of DLS of the entire lung was 2.6 (1.7–3.9). Absolute values of FEV1 and FVC were both negatively associated with DLS (*r* = −0.78, *P* = 0.001, and *r* = −0.61, *P* = 0.021). Percentage of predicted value of FEV_1_ and FVC were also negatively associated with DLS (*r* = −0.62, *P* = 0.019, and *r* = −0.76, *P* = 0.002). Our study shows that lung function of TDL patients were notably correlated with the extent of destroyed lung measured with chest CT scan.

## 1. Introduction

Tuberculous destroyed lung (TDL) is designated as a large destruction of lung parenchyma secondary to pulmonary tuberculosis. TDL often causes severe problems such as progressive dyspnea leading to irreversible respiratory impairment, repeated pulmonary infectious episodes, hemoptysis, and so on. It is known that these events develop about ten years later after onset of the initial disease [1]. Sequelae of chronic pulmonary tuberculosis may ensue, such as chronic bronchitis, bronchiectasis, emphysema, and fibrosis with chest wall retraction. In spite of the resultant damage of lung function and quality of lives of these patients, the exact mechanism or characteristics of pulmonary function worsening have not been fully understood. In contrast to other obstructive airway diseases, respiratory insufficiencies seen in TDL may therefore have a certain mechanism.

South Korea has been a tuberculosis endemic area in the past. A considerable portion of inadequate treatment cases of pulmonary tuberculosis resulted in destruction of lung parenchyma. As these patients grew older, TDL is now one of the causes of dyspnea of the elderly in South Korea [2]. To explain the correlation of TDL with diagnostic findings, some researchers adopted simple chest radiographs for predicting the airflow obstruction. To the best of our knowledge, however, there has been no study utilizing detailed investigational tools such as chest CT for interpreting the correlation. In this study, we investigated the feature of respiratory impairment in patients with TDL and also determined the correlation between the extent of destroyed lung measured with chest CT and lung function.

## 2. Methods

### 2.1. Study Subjects

Newly diagnosed TDL patients were enlisted in our study from January to December 2008. This study was approved by the Korea University Anam Hospital Ethics Committee, and all patients gave written informed consent. Patients who were stable without exacerbation of dyspnea were included. Exclusion criteria were as follows: positive sputum acid-fast bacilli smear, positive tuberculosis culture, or suspicious active tuberculosis on chest X-ray at presentation; coexisting medical problems, such as ischemic heart disease, asthma, chronic obstructive pulmonary disease, bronchiectasis, idiopathic pulmonary fibrosis, and malignancy; kyphoscoliosis or chest wall deformity/anomaly or history of chest surgery or major abdominal surgery; a smoking history of 20 pack years or more or a current smoker.

Patient demographics, tuberculosis treatment history, and comorbidities were all recorded. Pulmonary function test, chest radiograph, arterial blood gas (ABG) analysis, and chest CT were also performed. The diagnosis of TDL was based on a clear history of past tuberculosis with a finding of parenchymal destruction by tuberculosis verified by chest X-ray [3].

### 2.2. Pulmonary Function Tests (PFT)

Spirometry, lung diffusing capacity, and static lung volume were measured by body plethysmograph (Cardiopulmonary Function Test Vmax 229, Sensor Medics, Yorba Linda, CA, USA) according to recommendations in the Guidelines of the American Thoracic Society [4]. Obstructive pattern was defined by a reduced ratio of forced expiratory volume in 1 s (FEV_1_) to forced vital capacity (FVC) (FEV_1_/FVC ratio) below 70%. Restrictive pattern was defined by a reduction of total lung capacity (TLC) below 80% of the predicted value and also a normal FEV_1_/FVC ratio. Arterial partial pressure for O_2_ (PaO_2_) and CO_2 _(PaCO_2_) was measured in a standard anaerobic condition.

### 2.3. CT Examination

All images on chest CT were obtained as axial images using 16-detector row CT (Somatom Sensation 16; Siemens Medical Systems, Berlin, Germany). Chest CT with or without contrast enhancement was taken, and parameters were: 0.75 mm collimation, 5 mm slice thickness, 5 mm slice increment, B40 kernel, pitch 1.0, 120 kV, and 180 mAs. In case of contrast enhancement, a 130 mL amount of iodine contrast media (Ultravist 300, Bayer Healthcare, Berlin, Germany) was used with a 40-second delay after intravenous injection.

### 2.4. Visual Lung Assessment and Destroyed Lung Score (DLS) Calculation

Areas of destroyed lung were visually assessed by a chest radiologist (Y.W.O.) with a special interest in tuberculosis and nearly 30 years of field experience. The presence of calcified lesion, cavitary lesion, cicatrical lesion, and centrilobular lesion is confirmed as TDL lesion. Bronchiectatic lesion or emphysematous lesion was excluded.

**Figure 1:**
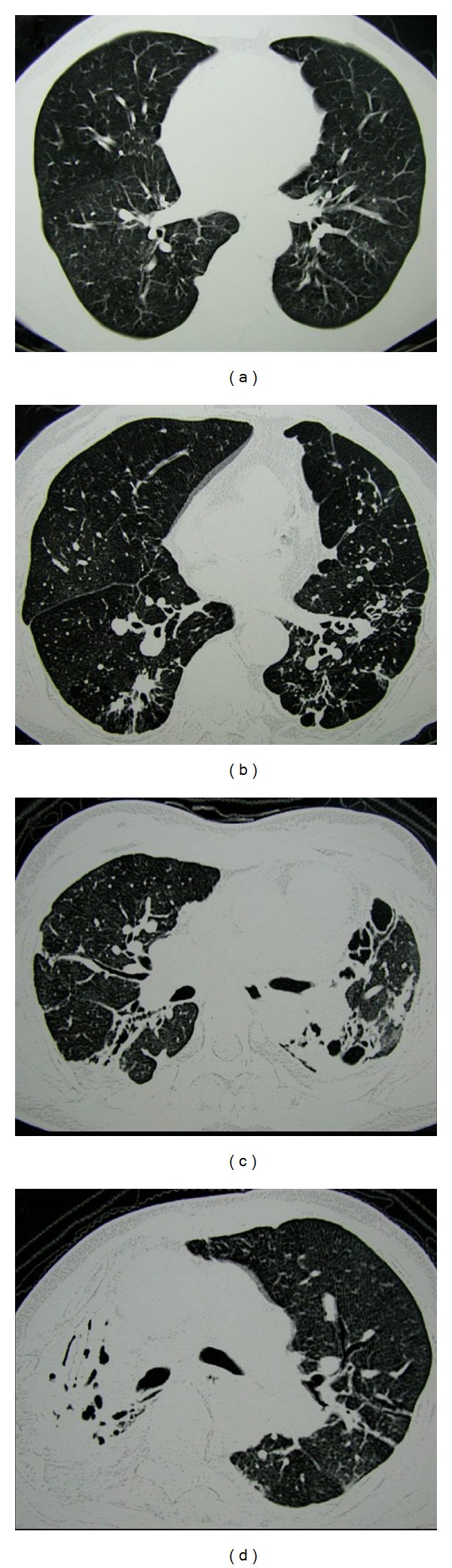
Some examples for grading the extent of TDL. (a) There was no abnormality, and a score of 0 was assigned. (b) A score of 1 was given, as less than 25% of the lung parenchyma showed destroyed lesion. (c) Destroyed lung lesion involved 50–75% of the lung parenchyma, and a score of 3 was assigned. (d) Destroyed lung lesion involved more than 75% of the lung parenchyma, therefore a score of 4 was assigned.

To briefly present the degree of destruction, we modified the Goddard classification scoring system [5] and developed a novel scoring system (destroyed lung score (DLS)). In detail, each slice was assessed, and right and left lungs were graded separately according to the percentage area showing destroyed lung. A score of 0 was assigned if there was no abnormality; 1 was given if less than 25% of lung parenchyma showed destroyed lung lesions; 2, 3, or 4 was assigned when destroyed lung lesions involved 25–50%, 50–75%, or more than 75%, respectively, of lung parenchyma (Figure 1). A DLS was then calculated by adding the scores for each slice and dividing by the total number of slices. The chest radiologist measuring and calculating DLS (E.J.C.) was blinded to patients' identity, clinical characteristics, and PFT results. 

### 2.5. Statistical Analysis

Statistical analysis was performed using computerized software (SPSS, version 13.0; SPSS Inc., Chicago, IL, USA). Normally distributed data were expressed as mean ± SD and skewed data as median. Spearman rank was used to assess nonparametric correlation. *P* < 0.05 was considered statistically significant

## 3. Results

Twenty-five subjects were enrolled; 17 were male, and 8 were female. Mean age was 62.6 ± 11.3 years. Three patients were judged to be cured after standard 6-month course of chemotherapy for tuberculosis. The rest of the patients (*n* = 22) has been treated for at least 3 years, maximally for 11 years because of repeated recurrences, multidrug resistance tuberculosis, or irregular medication with poor compliance.

Mean FEV_1_ and FVC were 0.89 ± 0.37 L (37.6 ± 14.1% predicted) and 1.72 ± 0.87 L (49.5 ± 19.9% predicted), respectively. Correspondingly, mean FEV_1_/FVC ratio was 57.0 ± 18.3%. Mean TLC was 105.3 ± 16.1%, while DLCO was 48.8 ± 22.7%. Sixteen patients showed an obstructive pattern; the number of patients with a restrictive pattern was 9. As for the ABG analysis, mean PaCO_2_ and PaO_2_ were 43.5 ± 12.6 and 63.0 ± 18.2 mmHg, respectively.

Data regarding the DLS are summarized on Table 1. Median value of DLS for the left lung was 1.0 (range 0.9–2.2) and the right lung 1.0 (range 0.8–1.7). Median value of DLS of the entire lung was 2.6 (1.7–3.9).

**Figure 2:**
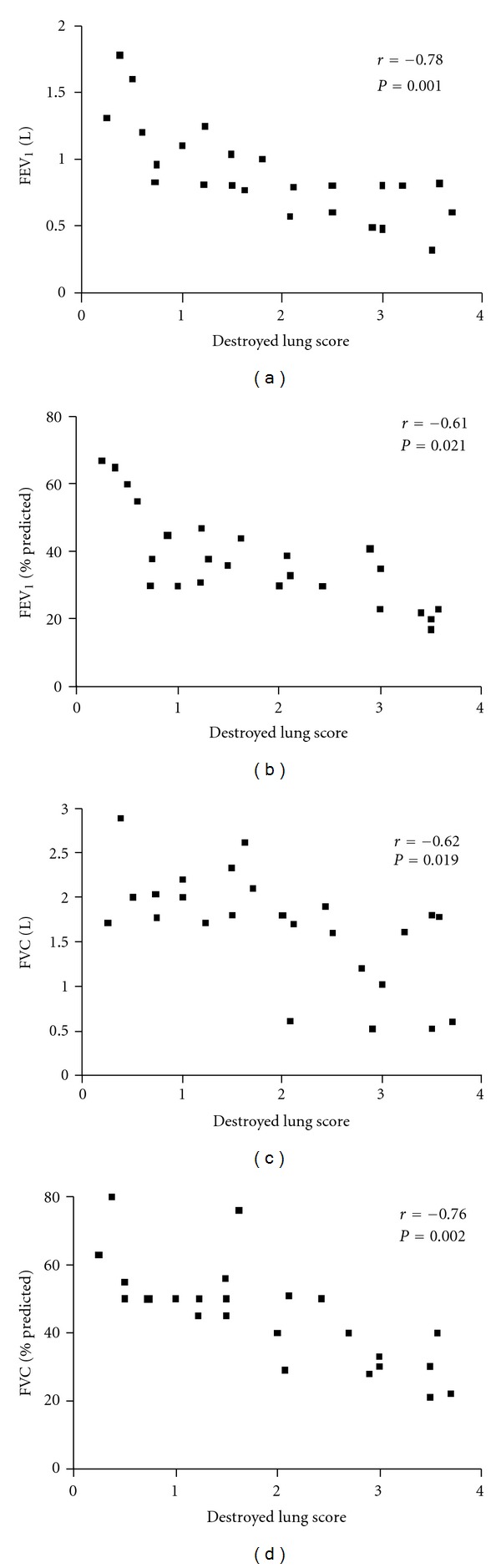
Illustrations showing close correlation between destroyed lung scores (DLS) and pulmonary function. (a) Relationship between DLS and FEV_1_ (L). (b) Relationship between DLS and FEV_1_ (%predicted). (c) Relationship between DLS and FVC (L). (d) Relationship between DLS and FVC (% predicted).

On analyzing the correlation of PFT and calculated scores obtained from chest CT, absolute values of FEV_1_ and FVC were both negatively associated with DLS (*r* = −0.78, *P* = 0.001 and *r* = −0.61, *P* = 0.021) (Figure 2). The percentage of predicted value of FEV_1_ and FVC were also negatively associated with DLS (*r* = −0.62, *P* = 0.019 and *r* = −0.76, *P* = 0.002) (Figure 2). However, FEV_1_/FVC was not associated with DLS (*r* = 0.17, *P* = 0.55) (Table 2).

## 4. Discussion

Results from our study show that TDL usually manifests as an obstructive pattern (64%, 16/25). We also proved that FEV_1_ and FVC both are negatively correlated with DLS from chest CT, suggestive of its usefulness for evaluating the extent of destroyed lung. Further, FEV_1_ and FVC showed a similar correlation with such destruction in both an obstructive and a restrictive pattern. It is common knowledge that FEV_1_ is chosen for an obstructive pattern and FVC for a restrictive one, to classify the disease according to its severity. Consequently, our result suggests that simple spirometric results may fulfill to accurately estimate the extent of lung destruction, when a CT image is not available.

In TDL, respiratory impairment originates from several anatomical features. For example, damage to bronchi resulting from extensive fibrosis or endobronchial stricture can cause airflow obstruction [6]. Greater lung volume loss appears to stem from parenchymal injury and subsequent fibrotic process [2]. In our study, an obstructive pattern was found in 64% of patients, and a restrictive pattern was evident in 36%. It is commonly assumed that an obstructive pattern is most likely due to an endobronchial injury, while a restrictive one to parenchymal damage; it is therefore pertinent to conclude that an obstructive pattern is the dominant feature of TDL based on our investigation. 

Several studies attempted to prove the correlation between simple chest radiography and lung function in tuberculosis patients. Williams et al. showed that the association between impairment of DLCO, but not vital capacity (VC), and the extent of abnormality on chest radiography was good in acutely ill, bedridden patients [7]. In this paper, however, the extent of involvement of tuberculosis in chest radiography was roughly estimated (i.e., the right upper lobe occupied 20%, right middle lung 10%, etc.). This might be inadvertently interpreted that there was no significant correlation between the extent of abnormality of chest radiography and VC. Another group showed that miliary tuberculosis was associated with diffuse restrictive lung disease, and DLCO can remain decreased despite complete clearing on the chest radiography [8]. However, this paper may be criticized that simple chest radiography may not exactly represent the extent of tuberculosis at all. Recently, one cohort study performed by Lam et al. found that the existence of inactive tuberculosis on chest radiography was associated with a higher risk of airflow obstruction, independent of smoking status [9]. Our study seemingly shows similar results indicating the utility of PFT on estimating the extent of TDL. However, simple radiographic examination fundamentally lacks detailed information of each TDL patient; it is clear that chest CT images prevail over chest X-rays in terms of examining and predicting various types of pulmonary diseases. According to our study, measuring PFT of TDL may lead to a better structured approach and meticulous therapeutic plan toward TDL patients. Our investigation may also give inspiration to researchers trying to diagnose and prevent the unwanted aftermath of many TDL patients, whose illness is intractable and clinical outcome is generally poor.

In conclusion, the present study is the first to show a relationship between lung function and extent of destroyed lung specifically using chest CT scan in patients with TDL. Currently, diagnostic criteria, clinical features, mechanism, treatment, and prognosis of TDL have not been clearly elucidated till date. Despite some limitations such as small number of patients, this study may add further knowledge for understanding TDL.

## Figures and Tables

**Table 1:** Clinical characteristics (*n* = 25).

Variables	
Age, yrs*	62.6 ± 11.3
Male/female	17/8

PFT results	
FEV_1_, L (% predicted)*	0.89 ± 0.37 (37.6 ± 14.1)
FVC, L (% predicted)*	1.72 ± 0.87 (49.5 ± 19.9)
FEV_1_/FVC, %*	57.0 ± 18.3
TLC, L (% predicted)*	105.3 ± 16.1
DLCO, mL/min/mmHg (% predicted)*	48.8 ± 22.7
Obstructive pattern/restrictive pattern	16 (64%)/9 (36%)

ABGA results	
PCO_2_, mmHg*	43.5 ± 12.6
PO_2_, mmHg*	63.0 ± 18.2

Extent of destroyed lung, score^†^	2.6 (1.7–3.9)

*Mean ± SD. ^†^median (range).

ABGA: arterial blood gas analysis, PFT: pulmonary function tests, FEV_1_: forced expiratory volume in 1 s, FVC: forced vital capacity, TLC: total lung capacity, DLCO: diffusing capacity for carbon monoxide.

**Table 2:** Correlation of destroyed lung scores with pulmonary function (Spearman rank correlation).

Variables	Correlation coefficient	*P* value
FEV_1_ (L)	−0.78	0.001
FEV_1_ (% predicted)	−0.62	0.018
FVC (L)	−0.61	0.021
FVC (% predicted)	−0.76	0.002
FEV_1_/FVC (%)	0.17	0.551

FEV_1_: forced expiratory volume in 1 s, FVC: forced vital capacity.
